# Accelerated partial breast irradiation in the elderly: 5-year results of high-dose rate multi-catheter brachytherapy

**DOI:** 10.1186/1748-717X-9-115

**Published:** 2014-05-16

**Authors:** Caroline Genebes, Marie-Eve Chand, Jocelyn Gal, Mathieu Gautier, Ines Raoust, Tarik Ihrai, Adel Courdi, Jean-Marc Ferrero, Isabelle Peyrottes, Jean-Michel Hannoun-Levi

**Affiliations:** 1 Department of Radiation Oncology, Antoine Lacassagne Cancer Center, University of Nice-Sophia, 33, avenue de Valombrose, Nice 06189, France; 2 Biostatistic unit, Antoine Lacassagne Cancer Center, Nice, France; 3 Department of Breast surgery, Antoine Lacassagne Cancer Center, Nice, France; 4 Department of Medical Oncology, Antoine Lacassagne Cancer Center, Nice, France; 5 Department of Pathology, Antoine Lacassagne Cancer Center, Nice, France

**Keywords:** Breast cancer, Elderly, Brachytherapy

## Abstract

**Objective:**

To evaluate clinical outcome after accelerated partial breast irradiation (APBI) in the elderly after high-dose-rate interstitial multi-catheter brachytherapy (HIBT).

**Methods and materials:**

Between 2005 and 2013, 70 patients underwent APBI using HIBT. Catheter implant was performed intra or post-operatively (referred patients) after lumpectomy and axillary sentinel lymph node dissection. Once the pathological results confirmed the indication of APBI, planification CT-scan was performed to deliver 34 Gy/10f/5d or 32 Gy/8f/4d. Dose-volume adaptation was manually achieved (graphical optimization). Dosimetric results and clinical outcome were retrospectively analyzed. Physician cosmetic evaluation was reported.

**Results:**

With a median follow-up of 60.9 months [4.6 – 90.1], median age was 80.7 years [62 – 93.1]. Regarding APBI ASTRO criteria, 61.4%, 18.6% and 20% were classified as *suitable*, *cautionary* and *non-suitable* respectively. Axillary sentinel lymph node dissection was performed in 94.3%; 8 pts (11.5%) presented an axillary involvement. A median dose of 34 Gy [32 – 35] in 8 to 10 fractions was delivered. Median CTV was 75.2 cc [16.9 – 210], median D90 EQD2 was 43.3 Gy [35 – 72.6] and median DHI was 0.54 [0.19 – 0.74]. One patient experienced ipsilateral recurrence (5-year local free recurrence rate: 97.6%. Five-year specific and overall survival rates were 97.9% and 93.2% respectively. Thirty-four patients (48%) presented 47 late complications classified grade 1 (80.8%) and grade 2 (19.2%) with no grade ≥ 3. Cosmetic results were considered excellent/good for 67 pts (95.7%).

**Conclusion:**

APBI using HIBT and respecting strict rules of implantation and planification, represents a smart alternative between *no post-operative irradiation* and *whole breast irradiation* delivered over 6 consecutive weeks.

## Introduction

With the increase in life expectancy, the incidence of breast cancer is growing in the elderly population. Whole breast irradiation (WBI), improving locoregional control and overall survival, remains the standard of care after breast conserving surgery, whatever the age of the patient [1-3]. The management of adjuvant radiation therapy in the elderly has become a medical and economic issue. Indeed, a total treatment time of 6–7 weeks has been shown to affect the observance [4] of WBI in the elderly patient sub-group mainly due to the high number of transportations. Moreover, it represents an important consumption of resources with the problematic of saturation of radiation therapy departments.

During the last decade, accelerated and partial breast irradiation (APBI) techniques have emerged, as an alternative to whole breast irradiation (WBI) for patients with early breast cancer and low risk of local recurrence [5-7]. The volume of breast tissue irradiated is smaller and the course of treatment is shortened, which is particularly interesting in the elderly [8]. Although consensus recommendations have been published (Bethesda workshop [9], ASTRO [10] and GEC-ESTRO [11]), APBI is not widely accepted as an alternative to WBI due to the lack of long follow-up results of the phase III trials having compared WBI vs APBI. Concerns remain about the risk of local recurrence requiring classically a salvage mastectomy.

Currently, different APBI techniques are used based on intraoperative (electrons or low-energy photons) or post-operative irradiation (brachytherapy or external-beam radiation therapy). Potential advantages of a specific technique over another are not clear, while the quality of the applied technique and the experience of the medical staff remain essential to achieve a good local control.

In this study, we investigated the results of a high-dose-rate (HDR) interstitial multicatheter brachytherapy (HIBT) as APBI in the elderly, in terms of clinical outcome.

## Material and method

### Patient features

From 2005 to 2013, 70 selected patients underwent a breast conserving surgery for early breast cancer. All along the study period, the 2004 Bethesda workshop and ASTRO recommendations were used for the selection of elderly women who could be good candidates for adjuvant APBI using HIBT [9,10]. However, for few frail patients presenting comorbidity factors, APBI was proposed without strong respect of the recommended APBI criteria. The protocol was approved by the central review board of the Antoine Lacassagne Cancer Center. Second conservative treatments in case of ipsilateral breast cancer recurrence were not considered.

### Breast surgery

Axillary dissection concerned Level I and II axillary lymph node area while sentinel lymph node biopsy alone was also achieved with extemporaneous exam and conversion to axillary dissection in case of positive biopsy. Then, lumpectomy was performed. Quality of margins was assessed by an extemporaneous pathological exam. Four to five clips were clamped by the surgeon to mark the tumor bed before closing the tumor bed cavity.

### Brachytherapy

Catheters (*Sharp Needles*™; Nucletron, an Elekta company, Elekta AB, Stockholm, Sweden) were inserted intra-operatively into the tumor bed according to the pre-operative mammogram data and the definition of the Clinical Target Volume (CTV) defined both by the surgeon and the radiation oncologist. The geometry of the implant was performed in respect to Paris system recommendations [12].

Once the final pathological results confirmed the indication of APBI, post-operative planification CT-scan was performed and the CTV was delineated taking into account a safety margin of 2 cm from the clips minus the surgical margins described by the pathologist in the 6 directions (latero-medial, antero-posterior, and cranio-caudal dimensions. The CTV was redefined as 5 mm below the skin-surface, and 5 mm above the underlying ribs for superficial and deep tumours respectively. In case of inadequate pathological features for APBI, brachytherapy was used as an anticipated-boost before WBI. The planification was performed using Plato™ then OncentraBrachy™ treatment planning systems (Nucletron, an Elekta company, Elekta AB, Stockholm, Sweden). Dose-volume adaptation was manually achieved using graphical optimization. Dose constraints were: D90 (dose delivered to 90% of the CTV) > 105%, V100 (part of the CTV receiving 100% of the prescribed dose) > 95%, V150 < 35%, V200 < 15% (with no confluence of two consecutive V200 isodoses and V200 isodose diameter < 10 mm) and DMskin (maximal dose delivered to the skin) < 75%. Two protocols were applied delivering 2 fractions per day (6 hours apart) up to a total dose of 34 Gy (3.4 Gy/fraction over 5 consecutive days) or 32 Gy (4 Gy/fraction over 4 consecutive days). Irradiation was performed with an after-loading device using a 10 Ci 192Ir source (Microselectron™; Nucletron, an Elekta company, Elekta AB, Stockholm, Sweden). The irradiation was performed in an out-patient hospitalization way.

### Systemic therapy

Adjuvant chemotherapy or hormonal treatments were proposed according to the protocols used in the Antoine Lacassagne Cancer Center.

### Follow-up

All patients were followed up closely. Clinical examination was performed 1 month after HIBT and then every 6 month (alternatively by the surgeon and the radiation oncologist. Mammograms were obtained yearly. Late toxicity was assessed according to CTCAE v.3 criteria [13]. Cosmetic results were assessed at every follow–up visit by the physician according to the Harvard criteria [14]: excellent (treated breast nearly identical to untreated breast), good (treated breast slightly different from untreated), fair (treated breast clearly different from untreated but not seriously distorted), and poor (treated breast seriously distorted). All patients were included in the follow-up. The median follow-up was calculated from the day of last brachytherapy fraction to the date of last follow-up.

### Statistical analysis

Data were analyzed using the R 3.0.1 Windows software. Quantitative data are represented as median, extreme, mean and standard deviation. Qualitative data are represented as frequency, percentage and confidence interval 95%.

Local recurrence-free survival (LRFS) was defined as the time between the date of surgery and the date of ipsilateral local recurrence. Metastatic disease free survival (MDFS) rate was defined as the time between the date of surgery and the date of metastatic disease occurrence. Specific survival (SS) and overall survival (OS) were defined as the time between the date of surgery and death from cancer or any cause respectively. These data were estimated and plotted at different time intervals with their 95% confidence using the Kaplan-Meier method. Patients were censored at the time of death or at last follow-up. The level of significance was set at a value of p less than 0.05.

## Results

### Patients and tumor characteristics

With a median follow-up of 60.9 months [4.6 – 90.1], median age was 80.7 years [62 – 9 3.1] while 90% of the patients were older than 70 (Table 1). According to the APBI ASTRO recommendations [10], 61.4%, 18.6% and 20% were classified as suitable, cautionary and unsuitable respectively. Among unsuitable patients, 1 patient underwent a neo-adjuvant hormonal therapy, 2 pts could not lift their arm (WBI was not technically feasible), 2 pts presented with a morbid obesity and underwent additional supraclavicular fossa EBRT because of their nodal status. Median tumor size was 11 mm [1.3 – 35], 8 pts (11.4%) presented with axillary lymph node involvement, 16 pts (22.8%) had a grade 3 tumor, hormonal status was negative (ER-/PR-) for 6 pts (8.5%) while Her2 status was considered as overexpressed for 6 pts (8.5%).

**Table 1:** Patients and tumor characteristics

**Characteristics**	**n**	**%/range**		
Median age	81.4	[62 – 93]		
APBI category				
Suitable	43	61.4		
Cautionary	13	18.6		
Non suitable	14	20		
Histological subtype				
IDC	61	87.1		
ILC	3	4.3		
OIC	1	1.4		
DCIS	5	7.1		
Median tumor size (mm)				
	11	[1.3 – 35]		
pN category				
pNx	4	5.7		
pN0	58	82.9		
pN1	7	10.1		
pN2	1	1.4		
Histological grading				
1	24	34.3		
2	28	40		
3	16	22.9		
unknown	2	2.8		
Hormonal status				
ER+/PR+	43	61.4		
ER+/PR-	14	20		
ER-/PR+	2	2.9		
ER-/PR-	6	8.6		
unknown	5	7.1		
Her2 status				
negative	55	78.6		
+	1	1.4		
++	3	4.3		
+++	6	8.6		
unknown	5	7.1		
LVI				
yes	3	4.3		
no	19	27.1		
unknown	48	68.6		
PNI				
yes	1	1.4		
no	14	20		
unknown	55	78.6		
Median Ki67 (%)				
12.5	[5 – 40]			
EIDC				
yes	5	7.1		
no	61	87.2		
unknown	4	5.7		
Clear margins				
	70	100		
Systemic therapy				
HT				
yes	56	80		
no	14	20		
CT				
yes	3	4.3		
no	67	95.7		

*IDC* invasive ductal carcinoma, *ILC* invasive lobular carcinoma, *OIC* other invasive carcinoma, *DCIS* ductal carcinoma in situ, *LVI* Lympho-vascular invasion, *PNI* Peri-neural invasion, *EIDC* Extensive intra-ductal component, *HT* Hormonal therapy, *CT* Chemotherapy.

### Brachytherapy technique and dosimetric results

Among the 70 patients, the majority (92.9%) was implanted per-operatively. Five referred patients were implanted post-operatively. Median time interval between surgery and brachytherapy was 12 days [5 – 105]. Median number of needles and plans were 8 |5 – 16] and 2 [1-3] respectively. Median total dose was 34 Gy [15-17] for a median number of fractions of 10 [8-10]. The median CTV was 75.2 cc [16.9 – 210]. Median EQD2 of the D90 using αβ = 4 was 43.3 Gy |35 – 72.6], median V100 was 95% [68.9 – 100] while median DHI was 0.54 [0.19 – 0.74]. Dosimetric results are summarized in Table 2.

**Table 2:** Technical and dosimetric data

**Data**		**Mean**	**Median**	**Interval**
^#^needles		9	8	[5 – 16]
^#^plans		2	2	[1 – 3]
CTV (cc)		82.3	75.2	[16.9 – 210]
Dose/f (Gy)		3.4	3.4	[3.4 – 4]
^#^fractions		10	10	[8 – 10]
Total dose (Gy)		34	34	[32 – 34]
Time interval S/B (d)		17.5	12	[5 – 105]
D90				
Gy		3.7	3.7	[2.2 – 8.4]
%		106.2	107.1	[64.1 – 125.9]
EQD2 (Gy)		43.6	43.3	[35 – 72.6]
D100				
Gy		2.6	2.7	[1 – 6.6]
%		74.8	77.5	[26 – 100.9]
EQD2 (Gy)		37.4	37.7	[26.9 – 59.8]
V100				
cc		76.4	72.2	[16.4 – 192]
%		93.3	95	[68.9 – 100]
V150				
cc		36.3	30.6	[7.4 – 96]
%		44.3	43	[22.1 – 81.2]
V200				
cc		13.6	11.2	[3.2 – 51.6]
%		17.1	16.6	[9.4 – 47.3]
DHI		52.8	53.6	[18.8 – 73]

^#^needles: number of needles; ^#^plans: number of plans; CTV: clinical target volume; Dose/f: dose per fraction; ^#^fractions: number of fractions; Time interval S/B: time interval between surgery and brachytherapy; D90: dose delivered to 90% of the CTV; D100: dose delivered to 100% of the CTV; V100: part of the CTV receiving 100% of the prescribed dose; V150: part of the CTV receiving 150% of the prescribed dose; V200: part of the CTV receiving 200% of the prescribed dose; DHI: Dose Homogeneity index (1-V150/V100); EQD2: equivalent dose at 2 Gy using an αβ = 4 for breast tissue and breast tumour.

### Local control and survival rates

**Figure 1:**
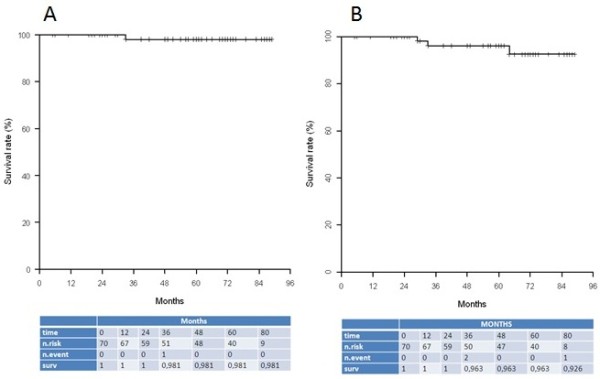
Kaplan-Meier curves for local recurrence free survival (A) and metastatic disease free survival (B).

One patient experienced an ipsilateral multifocal local recurrence (1.4%) associated with synchronous supraclavicular and metastatic relapses leading to a 5-year LRFS rate of 98.1% (95% CI [0.945; 1]) (Figure 1A). This 79 year-old patient belonged to the unsuitable group, underwent a neo-adjuvant hormonal therapy because of an initial metastatic disease doubt. The patient refused the chemotherapy proposed at the time of relapse but accepted hormonal therapy.

**Figure 2:**
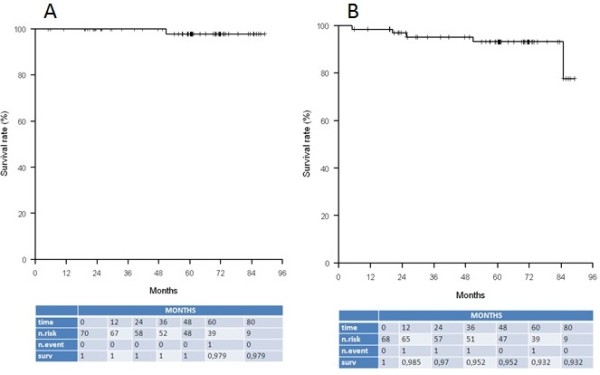
Kaplan-Meier curves for specific survival (A) and overall survival (B).

The 5-year MDFS rate was 96.3% ([95% CI [0.844; 1]) (Figure 1B). Taking into account the 79 year-old patient who presented a synchronous local and metastatic relapse, two additional patients developed a metastatic relapse. One belonged also to the unsuitable group (positive nodal status but she could not lift arms and additional supraclavicular EBRT was delivered). The second patient had an exclusive metastatic relapse and belonged to the suitable group. Characteristics of the patients who presented metastatic recurrences are detailed in Table 3.The 5-year SS and OS rates were 97.9% (95% CI [0.938; 1]) and 93.2% (95% CI [0.868; 0.999]) respectively (Figure 2A and B). Four patients (5.7%) died from other causes, one due to a head and neck cancer, tow due dementia, and one due to cardio-vascular disease.

**Table 3:** characteristics of patients with relapse

**Pt**	**ASTRO group**	**Type of relapse**	**Age at surgery**	**Histologic features**	**TTP (months)**	**Dose (Gy)**	**D90 (%)**	**V100 (%)**	**DHI**
1	Unsuitable	Local	79.2	IDC	32.9	34	103.2	92.2	0.44
		Regional		19 mm					
		Metastatic		pN0					
				grade 2					
				ER + PR-					
				HER2-					
2	Suitable	Metastatic	73.8	IDC	63.1	32	107	93.4	0.40
				18 mm					
				pN0					
				grade 1					
				ER + PR-					
				HER2-					
3	Unsuitable	Synchronous	83.5	IDC	27.3	34	69.7	75	0.44
		regional and		27 mm					
		metastatic		pN1					
				grade 3					
				HR-					
				HER2-					

IDC: invasive ductal carcinoma; TTP: time to progression; D90: dose delivered to 90% of the CTV; V100: part of the CTV receiving 100% of the prescribed dose; DHI: Dose Homogeneity index (1-V150/V100).

### Toxicity

Acute side effects consisted mainly in breast pain, edema or radio-dermatitis, while all the patients recovered spontaneously or with symptomatic medical management.

Thirty-four patients (48%) experienced at least one late toxicity. Among these complications, 38 (79.2%) were grade 1 and 9 (18.8%) grade 2. No grade 3 late side effects occurred. Late skin and breast toxicity consisted mainly in grade 1 sub-cutaneous fibrosis while dyspigmentation, breast deformation and telangiectasia were also observed. Toxicities are summarized in Table 4. Excellent or good cosmetic results were observed in 67 patients (95.7%).

**Table 4:** **Type and grade of late toxicity according to the CTCAE v.3 criteria **[13]

**Toxicity**	**Grade 1 (%)**	**Grade 2 (%)**
Dyspigmentation		
Hyper	4.3	0
Hypo	2.9	0
Telangiectasia	11	1.4
Breast deformation	5.7	0
Fibrosis		
Cutaneous	4.3	1.4
Sub-cutaneous	25.7	5.7

## Discussion

Breast conserving treatment (BCT) is the standard of care for early stage breast cancer. It consists of a conserving surgery followed by whole breast irradiation (WBI) up to a total dose of 45–50 Gy (delivered to the entire breast over 5 to 6 weeks with 1.8 to 2 Gy per fraction) with, in the majority of cases, a boost dose of 10–16 Gy to the tumor bed [1,15,16]. Radiation therapy is a mainstay of this conserving approach, not only allowing a threefold reduction in local recurrence but also improving overall survival [1-3]. Despite the advantages of BCT, adjuvant radiation therapy is sometimes underused for some reasons (convenience, patient age, distance from the radiation therapy center, lack of social support structure, physician bias and fear of radiation treatment). Studies have shown that 15% to 30% of patients undergoing lumpectomy do not receive a needed adjuvant radiation therapy [17,18]. This issue leads to undertreat patients or inversely to perform total mastectomy in patients who do not have access to adjuvant radiation therapy. Indeed, the usual 6–7 weeks course of irradiation lead to lack of observance and represents in many countries an issue of access and cost with the outsized consumption of resources in terms of patient visits, physician times and machines exploitation [19].

Regarding breast cancer in the elderly, this population appears negatively affected by age at diagnosis, clinical stage, and the presence of comorbidity factors [20,21]. Even if the incidence of breast cancer is growing in the elderly and its management has become a medical and economic issue, rates of radiation recommendation in the elderly have been steadily decreasing [22]. Recently, Hughes et al. [23] updated the results of a phase III trial (CALGB 9343) that randomized in the elderly who presented breast cancer (T1N0, HR positive) lumpectomy plus Tamoxifen with or without WBI. The authors confirmed that, at 10 years, post-operative breast irradiation improved loco-regional-free recurrence rate in women ≥ 70 year-old (98% vs 90% with or without WBI respectively; p < 0.001), but, this local control improvement did not translate into an advantage in overall survival, distant disease-free survival or breast preservation, considering that Tamoxifen alone was a reasonable exclusive option for women ≥ 70 year-old. On the other hand, it is well established that adjuvant breast irradiation significantly decreases the rate of local recurrence leading to a significant benefit in terms of breast cancer death [1-3]. Those results can be also observed in the elderly population in which Hancke et al. [24] described a damaging impact on OS and DFS in case of WBI omission. Regarding epidemiologic considerations, women aged 70 years and over who are currently in good health condition, have a median life expectancy of 15.5 years and half of them will live much longer and will remain exposed for enough time to the potentially preventable risks of a relapse and specific death [25]. Taking all together, those data lead to consider the adjuvant breast irradiation in the elderly as a specific and key question that remains under debate. Consequently, identifying a subset of women who may not benefit from the addition of radiation therapy after lumpectomy for early stage breast cancer has become an important issue but still unresolved [26].

In order to find an acceptable compromise between *no breast irradiation* and *5 to 6 weeks of WBI*, the concept of accelerated partial breast irradiation emerged and has been presented as a good option specifically for the elderly population with breast cancer at low risk of local recurrence [8]. Indeed, irradiation is limited to the higher risk area of local recurrence. By increasing the radiation fraction size and decreasing the target volume and consequently decreasing the volume of irradiated normal tissue, this technique allows a shorter treatment time, which is particularly interesting in the elderly leading to a potential increase of radiation observance. Many techniques of APBI have been developed: HIBT, balloon catheter brachytherapy, 3D-CRT (conformal radiation therapy) and intra-operative radiation therapy (IORT). HIBT is one the first APBI technique used and has the longest follow-up [27]. With a 12-year median follow-up, Polgar et al. [28] reported a 5-year LR rate of 4.7% with 77% of good/excellent cosmetic results. In 2005, based on the Besthesda workshop recommendations [9], we started an APBI HIBT program dedicated to elderly women. Later, ASTRO and ESTRO recommendations strengthened our indications [10,11]. In specific circumstances in which some eligibility criteria were missing, we were led to propose APBI for patients with particular features that make a classical EBRT impossible, such as functional disability to lift arms or morbid obesity. It was the case of 4 patients out of the 14 belonging to the ASTRO unsuitable group. With a median age of 81.4 years, our study is moreover representative of the specific elderly population. With a 2.4% 5-year local recurrence rate and 95.7% of excellent/good cosmetic results without any grade 3 adverse events, our study compares favorably with the literature data [28-30]. The only one patient who experienced an in-breast recurrence belonged to the ASTRO unsuitable group, and was not a good candidate APBI. It is essential to make a precise and rigorous selection of the eligible patients for APBI [31].

Although consensus recommendations from ASTRO [10] and GEC-ESTRO [11] have been published, the concept of APBI is not clearly validated without consistent results in terms of evidence based medicine. Beside the concept of APBI, it appears important to take into account the technique used for APBI. From now, four different studies were published focusing on APBI in the elderly. Three of them consisted in phase II prospective trials using either HDR brachytherapy based on a balloon device [32], IORT using electron beam radiation therapy [33] and HIBT [34]. All these studies confirmed that these different irradiation techniques were feasible with encouraging results in terms of clinical outcome. Furthermore, GERICO-03 study specifically analyzed the functional status after APBI and concluded that HIBT had no deleterious impact in the elderly [34]. The fourth study was a non-randomized retrospective one recently published by Smith et al. [35]. In a cohort of elderly women with breast cancer (mean age of 74.8), the authors compared APBI with brachytherapy and WBI and reported worse long-term breast preservation and increased complication rates for patients treated with brachytherapy without any significant impact on survival. The author advised “prompt caution over widespread application of breast brachytherapy”. This assertion is acceptable if we consider brachytherapy technique based on a balloon device (Mammosite™) but not if HIBT is used [36-38]. Recently, the same authors concluded, in an observational study, that brachytherapy shows lesser breast preservation benefit compared with EBRT and that the ASTRO suitability criteria identify patients with the lowest absolute, but not relative, risk of mastectomy [39]. However, it was a methodologically objectionable observational study, using again a single-lumen balloon applicator. Polgár et al. [40] published the updated results at 10 years of a phase III randomized trial comparing APBI using HIBT versus WBI for a selected group of patients with early stage breast cancer. They authors reported similar 10-year results between the two arms in terms of local control while significantly better cosmetic outcome was observed in the HIBT arm. Furthermore, the GEC-ESTRO Breast Cancer Working Group recently reported the clinical outcome of a 2nd conservative treatment based on lumpectomy plus HIBT in case of ipsilateral breast tumor recurrence [41]. The authors noticed that, even in a context of accelerated partial breast re-irradiation, late side effects were mainly grade1/2 (rate of grade 3–4 = 11%) while excellent/good cosmetic result was achieved in 85% of the patients.

The weaknesses of the present study are mainly represented by the retrospective status of this analysis and the small number of patients but also, the very small number of events that did not allow performing uni/multivariate analysis researching prognostic factors for relapse or side effect. Furthermore, it was not possible to compare the 3 groups at risk (suitable, cautionary and unsuitable) according to clinical outcome especially for local recurrence rate.

Nevertheless, this study contributes to point out the impact of the APBI technique used on clinical outcome. Indeed, before going forward in the accurate analysis of the phase III randomized trials that started to emerge, it is important to keep in mind that the definitive validation (or not) of the APBI concept will be probably strongly correlated to the technique used to achieve this specific breast irradiation.

## Conclusion

While there is probably a sub-group of elderly women who does not need any post-operative radiation therapy for achieving good local control, this specific sub-group is currently not well defined. So, for the elderly and regarding clinical outcome, high-quality APBI using HIBT and respecting implantation rules, represents a smart alternative between no post-operative irradiation and whole breast irradiation delivered over 6 consecutive weeks.

### Consent

Written informed consent was obtained from the patient for the publication of this report and any accompanying images.

## Competing interests

The authors declare that they have no competing interests.

## Authors’ contribution

CG analyzed the clinical data and wrote the manuscript; MEC analyzed the clinical data and revised the “Brachytherapy” section; JG managed the statistical analysis; IR revised the “Breast surgery” section; TI revised the “Breast surgery” section; AC analyzed the clinical data and revised the “Discussion” section; JMF revised the “Discussion” section; IP analyzed the pathological samples; JMHL conceived, planned the study and wrote the manuscript. All authors read and approved the final manuscript.
